# Phenotype Shift from Atypical Scrapie to CH1641 following Experimental Transmission in Sheep

**DOI:** 10.1371/journal.pone.0117063

**Published:** 2015-02-24

**Authors:** Marion M. Simmons, S. Jo Moore, Richard Lockey, Melanie J Chaplin, Timm Konold, Christopher Vickery, John Spiropoulos

**Affiliations:** Animal and Plant Health Agency—Weybridge, Woodham Lane, Addlestone, Surrey, KT15 3NB, United Kingdom; The Scripps Research Institute Scripps Florida, UNITED STATES

## Abstract

The interactions of host and infecting strain in ovine transmissible spongiform encephalopathies are known to be complex, and have a profound effect on the resulting phenotype of disease. In contrast to classical scrapie, the pathology in naturally-occurring cases of atypical scrapie appears more consistent, regardless of genotype, and is preserved on transmission within sheep homologous for the prion protein (*PRNP*) gene. However, the stability of transmissible spongiform encephalopathy phenotypes on passage across and within species is not absolute, and there are reports in the literature where experimental transmissions of particular isolates have resulted in a phenotype consistent with a different strain. In this study, intracerebral inoculation of atypical scrapie between two genotypes both associated with susceptibility to atypical forms of disease resulted in one sheep displaying an altered phenotype with clinical, pathological, biochemical and murine bioassay characteristics all consistent with the classical scrapie strain CH1641, and distinct from the atypical scrapie donor, while the second sheep did not succumb to challenge. One of two sheep orally challenged with the same inoculum developed atypical scrapie indistinguishable from the donor. This study adds to the range of transmissible spongiform encephalopathy phenotype changes that have been reported following various different experimental donor-recipient combinations. While these circumstances may not arise through natural exposure to disease in the field, there is the potential for iatrogenic exposure should current disease surveillance and feed controls be relaxed. Future sheep to sheep transmission of atypical scrapie might lead to instances of disease with an alternative phenotype and onward transmission potential which may have adverse implications for both public health and animal disease control policies.

## Introduction

Scrapie belongs to a group of diseases known as the transmissible spongiform encephalopathies (TSEs) or prion diseases. Prions are believed to be composed exclusively of abnormal isoforms, designated PrP^Sc^ of a host encoded cell membrane bound protein, designated PrP^C^. The occurrence of different prion ‘strains’ is inferred from consistent differences in disease phenotype—the observable clinical presentation, pathogenesis, pathology and PrP molecular characteristics of the diseased host—and the disease phenotype observed in wild type or PrP transgenic rodent models. The one factor which is consistent in all cases, and forms the principal component of the current statutory diagnostic methods for TSE, is the disease-specific accumulation of PrP^Sc^ as demonstrated by immunochemical techniques such as ELISA, immunohistochemistry or Western blotting.

Small ruminants are affected by at least two forms of naturally occurring scrapie which have different molecular and neuropathogical characteristics. Classical scrapie has been reported as a clinical entity in sheep for at least 250 years. Following experimental challenges distinct ‘strain’ characteristics can be identified [1], but when naturally-occurring disease is studied it is clear that the amino acid PrP sequence of the host can have a significant effect on the resulting phenotype [2,3] although a single genotype may support more than one phenotype [2–4].

A distinctly different phenotype of ovine TSE, termed atypical scrapie (or Nor98) has recently been recognised, although it is speculated to have existed undetected for much longer than this [5,6]. It is found at a very low prevalence throughout the global sheep population including populations which have never recorded a case of classical scrapie such as New Zealand [7] and Australia (http://www.animalhealthaustralia.com.au/wp-content/uploads/2011/03/Australia-and-New-Zealand-Standard-Diagnostic-Protocols-for-TSE.pdf). In general atypical scrapie occurs as a single case on a farm, usually in older animals (over 5 years of age) and rarely presents with clinical signs that would be considered indicative of TSE [8]. It is only since the introduction of systematic active surveillance of healthy slaughter and fallen stock animals that these cases have been identified. In contrast to the widespread variation in histopathology and immunopathology characteristics seen in classical scrapie [2,3], the pathology observed in naturally occurring atypical scrapie cases appears to be much more consistent, regardless of genotype [9–12]. Atypical scrapie isolates can be transmitted experimentally through intracerebral inoculation into ovinised transgenic mice [13,14] and sheep, by either the intracerebral [8,15] or the oral route [16], with retention of the agent properties. However, epidemiological analysis of atypical scrapie cases supports the view that this TSE is not contagious, or has a very low transmissibility under natural conditions suggesting a sporadic or genetic origin [17–20].

There are remarkable similarities and differences between atypical and classical scrapie. Most notably, in sheep they both show affiliation to specific PrP genotypes, but breeding for resistance to classical scrapie enriches for genotypes that are susceptible to atypical scrapie and vice versa. As an example, valine (V) at codon 136 of the *PRNP* gene confers susceptibility to classical and resistance to atypical scrapie; conversely, arginine (R) at codon 171 confers resistance to classical and susceptibility to atypical scrapie, and histidine (H) at 154 is associated with susceptibility to atypical scrapie [21,22]. There is, however, some overlap in susceptible genotypes. Indeed, classical and atypical scrapie have been reported to co-exist in a single A_136_R_154_Q_171_/ARQ sheep [23]. The complex interactions between host and agent which determine the overall phenotype in a given animal are not yet understood.

The stability of TSE isolates on passage across and within species is not absolute, however, and there are increasing numbers of reports in the literature where experimental transmissions of particular isolates have resulted in a phenotype consistent with a different strain [24–27]. Indeed it has been postulated for decades that some alteration to the scrapie agent within the meat and bone meal rendering process was initially responsible for the BSE epidemic [28]. As new forms of TSE such as atypical scrapie and atypical (H- and L-type) bovine spongiform encephalopathy (BSE) are identified, speculation has increased about whether one of these potentially long-standing but rare strains could be the origin of BSE [24].

Despite naturally-occurring atypical scrapie being observed in a range of genotypes, successful experimental transmissions of clinical disease have so far only been reported within a particular homologous donor-recipient genotype model using sheep which are AHQ/AHQ homozygous [8,15,16]. These published transmissions represent part of a large study at APHA which has been running since 2004, investigating the potential transmissibility of atypical scrapie in a range of both homologous and cross-genotype combinations. Here we describe an unexpected and interesting finding from that study where one experimental challenge in which atypical scrapie from an ARR/ARR donor was inoculated intracerebrally into two AHQ/AHQ recipient sheep, and in one of them the resulting disease had a phenotype that was indistinguishable from CH1641 [29], a classical scrapie strain which has some BSE-like Western blot properties.

## Materials and Methods

### Ethics statement

All intracerebral inoculations were carried out under general anaesthesia, and in accordance with the United Kingdom (UK) Animal (Scientific Procedures) Act 1986, under Licence from the UK Government Home Office (Project licence number 70/5780 for the sheep and 70/7159 for the mouse challenges). Such licences are only granted following approval by the internal APHA ethical review process as mandated by the Home Office.

### Experimental challenges in sheep

The sheep described in this paper were challenged as part of a larger study that included in total seven donor and 54 recipient animals (excluding controls) to investigate the transmissibility and phenotypic stability of atypical scrapie in both homologous and heterologous donor/recipient genotype combinations. All recipient sheep were sourced from Defra’s New Zealand-derived flock, which is free from classical scrapie [30].

Although this study is still ongoing emerging data have already been published with regard to successful homologous AHQ/AHQ transmissions [8,15]. Donor animals were all naturally-occurring field cases, from which limited material was available. For the six animals reported in this paper, inoculum was prepared from the brain of a single naturally occurring case of atypical scrapie in an ARR/ARR sheep which was characterized initially by immunopathology and Western blot and subsequently strain typed in an ovine transgenic mouse model (tg338) as described [14].

Two VRQ/VRQ and two AHQ/AHQ sheep were challenged orally with 5g of neat brain homogenate [31]. Inoculum (10% w/v in normal saline) prepared from the same donor was microbiologically cleared by the addition of gentamycin (Hospira UK Ltd) at 0.25mg per ml. One ml of infected brain homogenate was inoculated intracerebrally into the thalamic region in each of two AHQ/AHQ recipient sheep [31].

All challenged animals were euthanized when they presented with clinical disease, or kept until seven years post challenge, as a pre-determined study endpoint. At post mortem, the whole brain was removed and hemisected longitudinally. One half of the brain was placed into 10% formal saline for histology, and the other half stored at -80°C. A range of lymphoid tissues were also collected.

### Experimental challenges in tg338 mice

An aliquot of the same inoculum that was used for the intracerebral challenge of the two AHQ/AHQ sheep, the frontal cortex of the positive AHQ/AHQ recipient and frontal cortex from a CH1641 experimentally challenged sheep were screened for microbiological sterility prior to inoculation into mice. Panels of 10 tg338 transgenic mice which over-express an ovine VRQ *PRNP* allele on a murine *PRNP* null background and are known to be susceptible to both atypical and classical scrapie [13] were inoculated intracerebrally with 20μl and intraperitoneally with 100μl of homogenate. Mice were monitored weekly and were killed when they had shown clinical signs on two out of three consecutive monitoring days, or at natural lifespan. Brains were then fixed, processed and lesion profiles produced as described in detail elsewhere [32,33].

### Clinical assessment of sheep

A neurological examination and behavioural observations were conducted when animal care staff suspected clinical disease (for more details, see [15]).

### Histopathology & Immunohistochemistry

Ovine brain tissue was routinely fixed, processed into paraffin wax, sectioned and stained with haematoxylin and eosin as described in detail elsewhere [2]. Immunohistochemical detection of PrP^Sc^ was performed as previously described [15] using mouse monoclonal antibody 2G11 (ABD Serotec), raised against ovine PrP peptide sequence 146- R_154_ R_171–_182, and specifically recognising the R_151_-R_159_ sequence. Tissues from the lymphoreticular system were immunolabelled using the same method. Brain immunolabelling was recorded and analysed using a ‘short PrP^Sc^ profiling protocol’ as described [11].

Murine brains were sectioned parasagitally and the larger portion (approximately 2/3 of the brain) was fixed in 10% buffered formalin and processed for histological examination. Sections (3μm thick) were stained with haematoxylin and eosin, and the intensity of vacuolation was assessed semi-quantitatively at specific neuroanatomical areas and used to produce lesion profiles as described previously [33,34].

Immunohistochemical detection of PrP^Sc^ was performed according to standard methodology using mouse monoclonal antibody R145 (APHA, Weybridge, UK), which recognizes amino acids 221–233 of bovine PrP [32]. The smaller portion of the brain was frozen at -80°C for subsequent biochemical or biological characterization.

### Western blotting

Unfixed brain samples were subjected to the TeSeE Universal WB (Bio-Rad Cat No: 355 1169 Marnes-la-Coquette, France) as previously described [15]. The sample from the donor animal required a double quantity of tissue extraction to improve the signal strength for clear profile classification. Additional Western blotting was performed following deglycosylation using PNGase and detection with mAb SAF84 conjugated to horseradish peroxidase. This protocol detects the presence of an additional band, in the region of 14kD, that is associated with CH1641 scrapie strain and is described in detail by Baron et al [35].

### Comparative data

Following initial observations that one challenged animal displayed unexpected characteristics which were similar to the classical scrapie strain CH1641 [36], unpublished observations from other experimental challenges with known CH1641 were retrospectively collated for comparative control purposes. These challenges included materials experimentally generated at APHA by intracerebral inoculation of AHQ/AHQ sheep with a reference CH1641 isolate kindly supplied by the TSE Resource Centre, Roslin Institute & R(D)SVS,University of Edinburgh,

## Results

### Oral challenges

The two VRQ/VRQ sheep, and one of the AHQ/AHQ sheep, orally challenged with the ARR/ARR atypical inoculum were clinically normal at the end of the seven year challenge period, and were negative by all post mortem tests.

During the pre-cull clinical examination at seven years post challenge, the other AHQ/AHQ sheep displayed mild ataxia, a wide-based hind gait and a fine head tremor. It was also teeth grinding. When blindfolded, it tended to circle to the left and displayed loss of balance. After the examination it aimlessly circled to the right in wide circles. It appeared to be confused and did not immediately follow other sheep (S1 File). Three cases of atypical scrapie have arisen within the Defra NZ-derived flock since these experimental challenges took place [30], but they were all in ARQ/ARQ sheep.

Pathologically, there was no detectable immunolabelling at the obex, and quite restricted labelling in the cerebellum and remaining brainstem. Cortical labelling was very diffuse, but widespread, as was the labeling within the basal ganglia. The clinical and pathological features of this animal, and the Western blot profile, were all consistent with atypical scrapie, including the absence of any detectable PrP^Sc^ in any lymphoid or peripheral nervous system tissues (data not shown).

### Intracerebral challenges

One sheep remained clinically normal until it was culled at seven years post challenge, and was TSE negative by all post mortem tests.

The other sheep presented with acute onset clinical abnormalities at 514 days post inoculation (dpi). It was found in lateral recumbency by animal care staff who suspected an epileptic fit. Following this period of recumbency, the animal was reported to be circling or drifting to one side. These observations could not be confirmed during systematic neurological examination, which would be compatible with them being transient post-ictal signs, but the animal was found to have visual impairment (bilateral absent menace response, collision with an object on eye level although it appeared to be able to negotiate obstacles on the ground) and hind limb ataxia (see movie PG 0306–08.mov). There was no wool loss suggestive of pruritus and it did not respond to scratching of the back (negative scratch test response). Subsequent CCTV observation indicated that the animal had difficulty lying down and standing up and was less active, standing occasionally in the pen with its head lowered, but no pruritic behavior was displayed. It was euthanized at 515 dpi.

These clinical signs were similar to those exhibited by sheep challenged with experimental CH1641 (n = 4) which, had a mean incubation period of 463±2 dpi, displayed ataxia but had no evidence of pruritus and some also lacked a menace response (Konold, unpublished data). They were also similar to the most consistent clinical signs evident in AHQ/AHQ sheep intracerebrally inoculated with atypical scrapie brain from AHQ/AHQ sheep (ataxia, and absence of both a menace response and pruritus), although average incubation periods were longer and more variable (range 378–1057), at 758±208 dpi (n = 9) [8].

**Fig 1:**
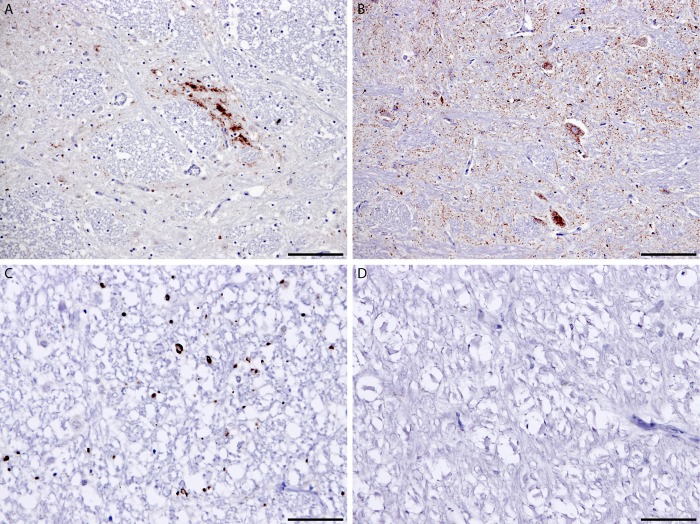
Immunohistochemistry of brain in donor and recipient sheep. Trigeminal nucleus from donor (A) and recipient (B) animals; intraneuronal PrP^Sc^ is evident only in the recipient animal (B). White matter from donor (C) and recipient (D) animals; globular white matter labelling is evident only in the donor animal (C). Scale bar in (A) and (B) indicates 100μm; in (C) and (D) indicates 50 μm. Antibody 2G11

**Fig 2:**
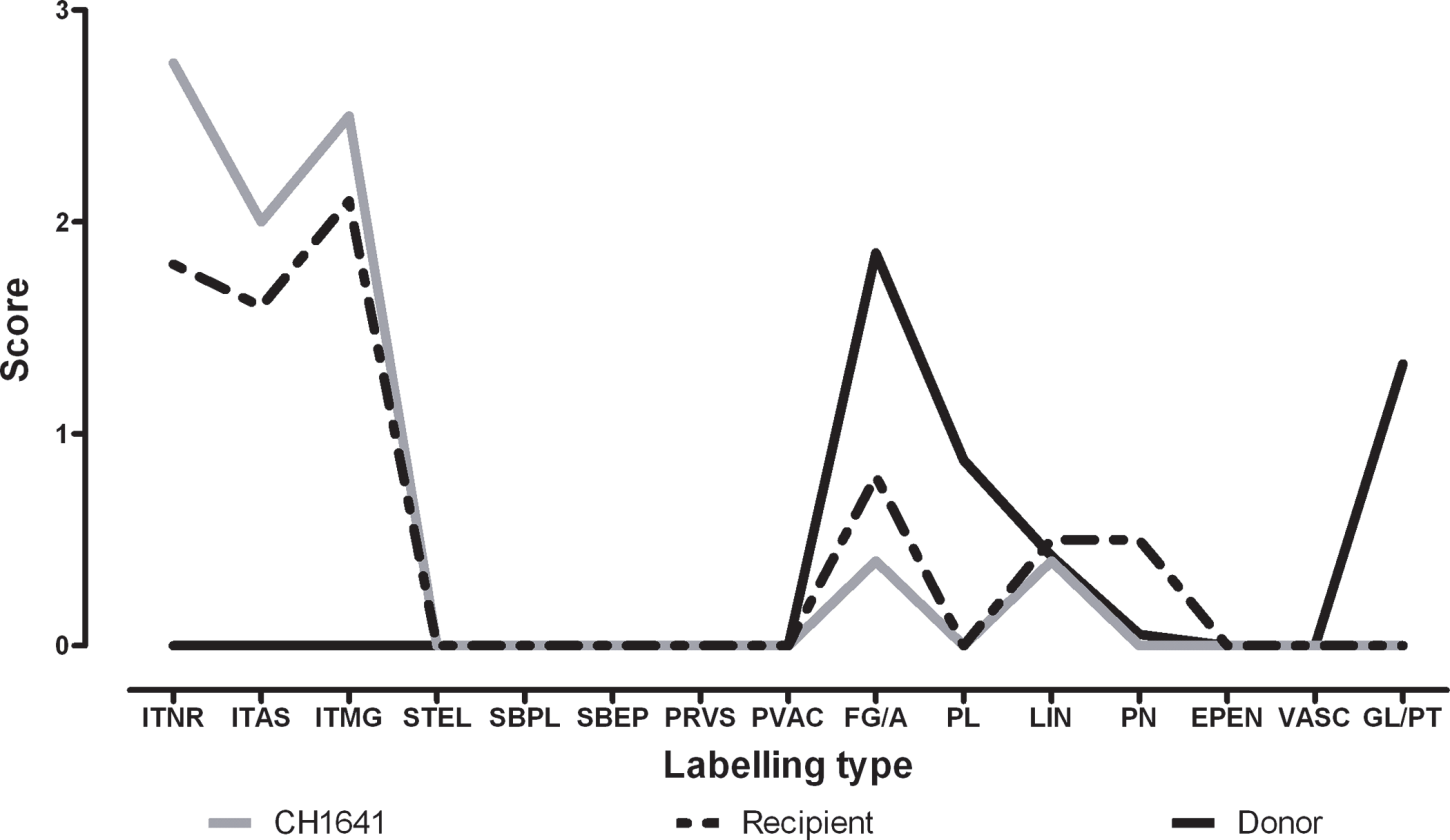
PrP^Sc^ profiles from donor, recipient and reference CH1641 at the level of the basal nuclei. Immunolabelling types: ITNR intraneuronal, ITAS intra-astrocytic, ITMG intramicroglial, STEL stellate, SBPL subpial, SBEP subependymal, PRVS perivascular, PVAC perivacuolar, FG/A fine granular and aggregates, PL plaque-like aggregates, LIN linear, PN perineuronal, EPEN ependymal, VASC vascular plaques, GL/PT globular and punctuate.

The immunolabelling seen throughout the neuraxis in this sheep was predominantly intracellular, in addition to the particulate labeling of the neuropil. (Fig. 1). The PrP^Sc^ short protocol mapping resulted in a profile that was indistinguishable from that of CH1641 [11] (Fig. 2) and quite different from that obtained from cases of atypical scrapie. This labelling is distinct from that resulting from other known TSE in this genotype of sheep [11].

**Fig 3:**
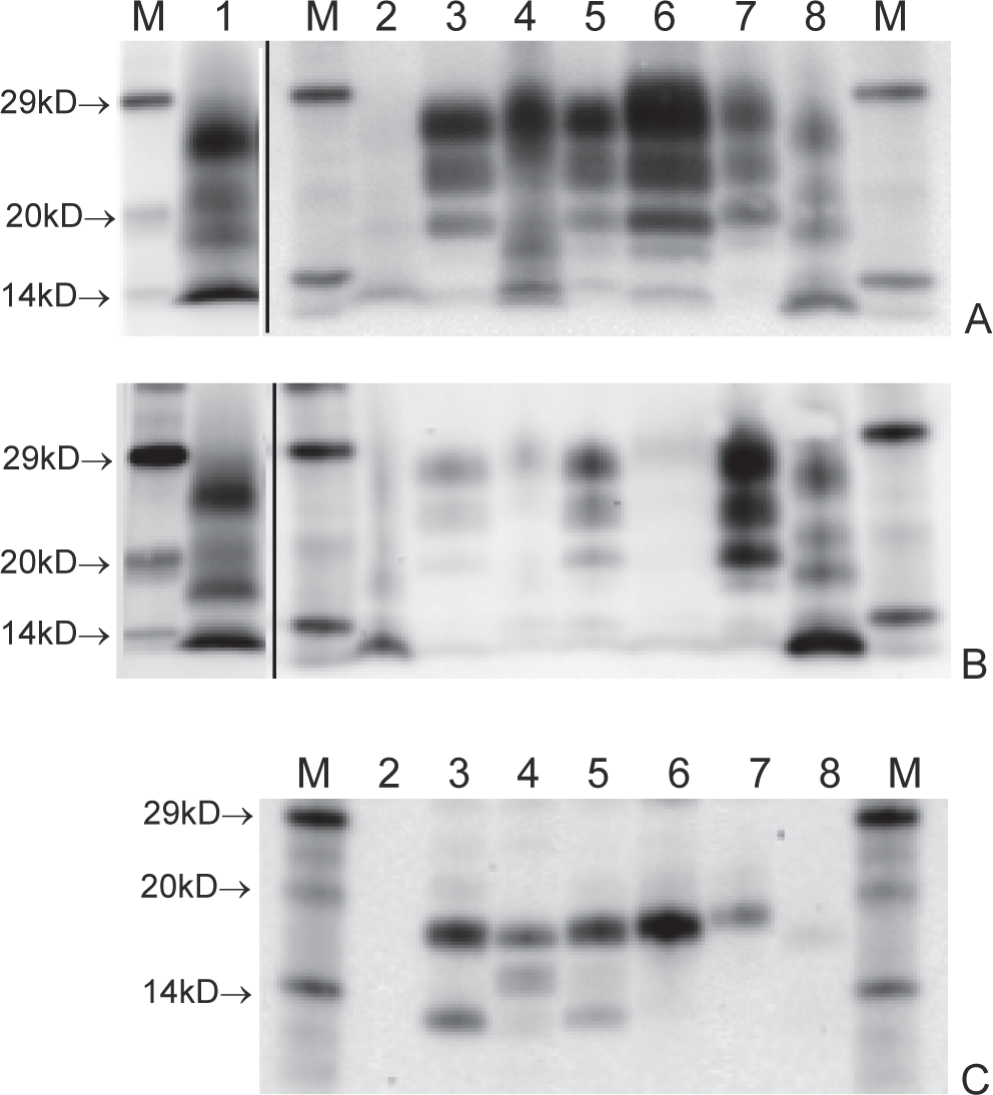
Western blot profiles. Following detection with mAb SHA31 (A); mAb P4 (B) and mAb SAF84 following de-glycosylation (C) Western blots show the atypical scrapie profile characteristics of the donor compared to the CH1641-like profile for the recipient. Lane key: M = molecular mass markers; 1 = donor (concentrated inoculum); 2 = donor (brain tissue); 3 = recipient; 4 = ovine BSE; 5 = ovine CH1641 scrapie; 6 = bovine BSE; 7 = ovine classical scrapie; 8 = ovine atypical scrapie.

The Western blot profile obtained from this sheep exhibited a three band pattern with a low molecular mass migration, relative to classical scrapie, when detected with mAb SHA31. When detected with mAb P4, it also had a much reduced signal intensity compared to the classical scrapie control, which was similar to the reduction in signal observed in the ovine BSE and CH1641 positive controls. In contrast, the profile observed in the brain tissue from the donor corresponded to that of the atypical scrapie control, originally at a weaker intensity but clearly visible in the concentrated sample (Fig. 3A, 3B).

Following deglycosylation and detection with mAb SAF84 the presence of a band at approximately 12kD was observed, the same as detected in the CH1641 control but not in the ovine BSE or bovine BSE control (Fig. 3C).

Overall, the WB results characterized the donor as atypical scrapie and the recipient as CH1641-like scrapie.

### Experimental challenges in tg338 mice

**Fig 4:**
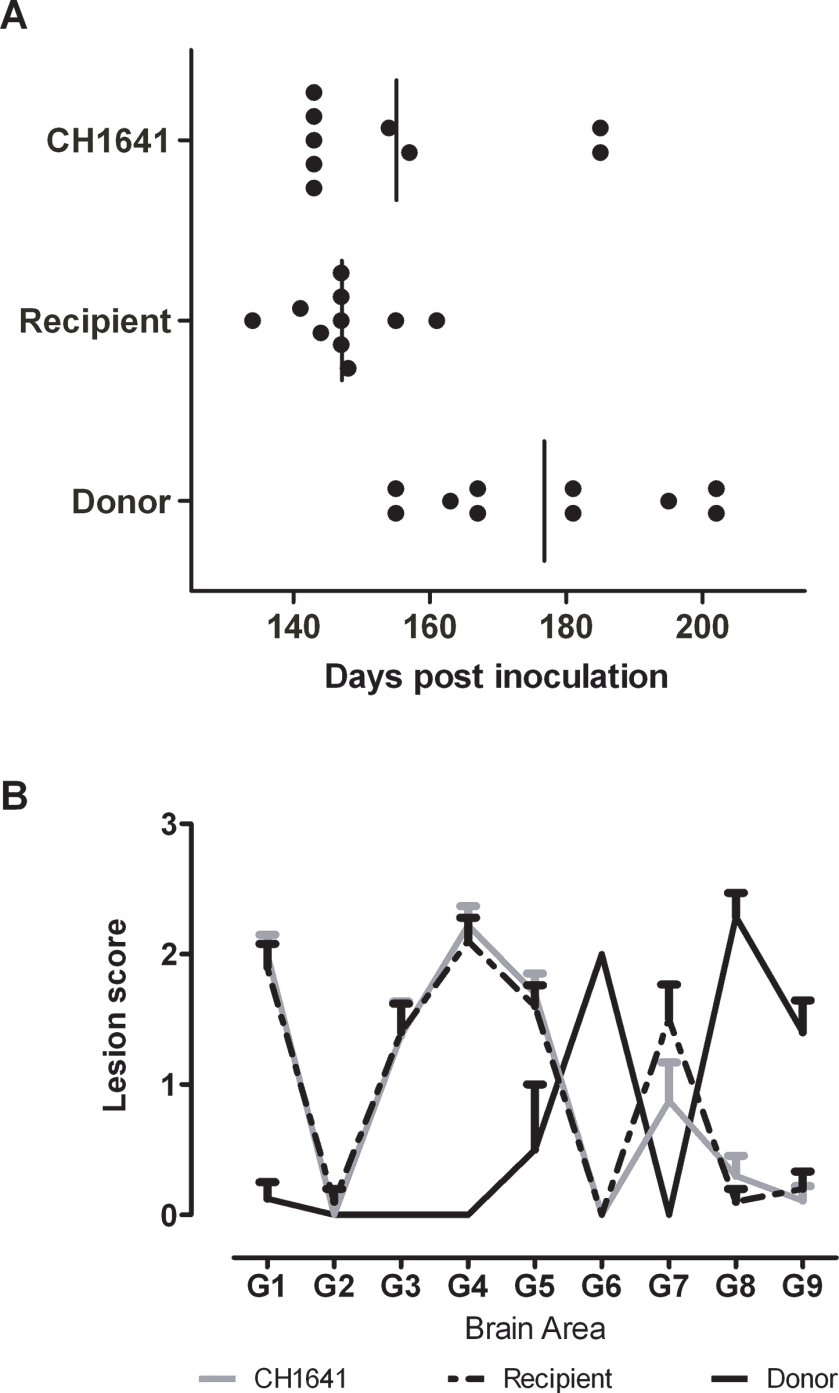
Bioassay data from Tg338 mice challenged with sheep brain. Incubation period (A) and lesion profile (B) from tg338 mice challenged with donor, recipient or reference CH1641 sheep. The data shown represent clinically and histopathologically positive mice. Profiles were obtained following quantitation of TSE specific vacuolation in nine neuroanatomic grey-matter areas (G1, dorsal medulla nuclei; G2, cerebellar cortex of the folia, including the granular layer, adjacent to the fourth ventricle; G3, cortex of the superior colliculus; G4, hypothalamus; G5, thalamus; G6, hippocampus; G7, septal nuclei of the paraterminal body; G8, cerebral cortex (at the level of G4 and G5); G9, cerebral cortex (at the level of G7)) and three white matter areas (W1, cerebellar peduncles; W2, white matter in lateral tegmentum;W3, cerebral peduncle).

**Fig 5:**
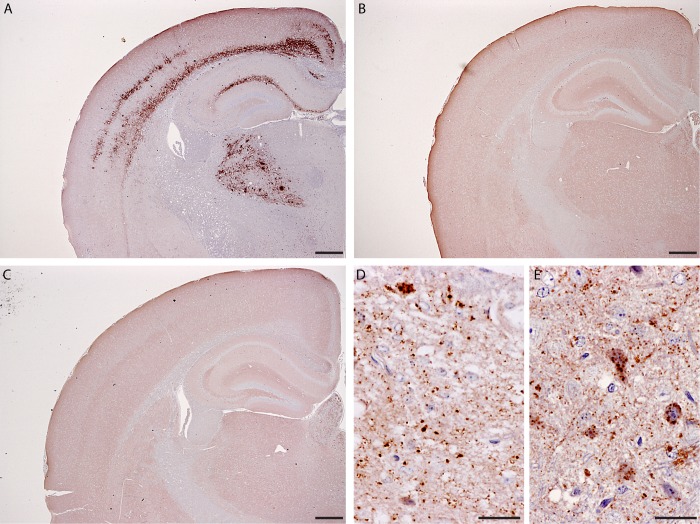
Immunohistochemisty of brain from challenged mice. Coronal sections at the thalamic level showing cerebrum, hippocampus and thalamus from mice inoculated with cerebral cortex from donor (A), recipient (B) and reference CH1641 (C) sheep. Mice inoculated with recipient (B) and reference CH1641 (C) material show no prominent PrP^Sc^ in these areas at this magnification. They do, however, show strong intracellular and neuropil PrP^Sc^ in other areas such as the medulla as shown in (D) and (E). Scale bar in (A)–(C) indicates 250 μm; in (D) and (E) indicates 25 μm. Antibody R145

The incubation period data obtained from bioassay of the donor sheep into tg338 mice were compatible with those obtained from atypical scrapie and differed from those obtained from the recipient sheep which in turn were similar to the ones produced by a reference CH1641 isolate (Fig. 4A). The mouse lesion profile obtained from the recipient animal (Fig. 4B) was very similar to that of a reference CH1641 inoculum in tg338 mice, and completely different to that of the donor, which had a profile consistent with atypical scrapie in Tg338 mice [13,14]. Mapping of PrP^Sc^ types in the mouse brain was in agreement with the incubation period and lesion profile data (Fig. 5).

## Discussion

It is clear from all the different aspects of phenotype investigated in this study that one recipient sheep in this experimental transmission expressed a phenotype that was CH1641-like, and different from that of the donor which was diagnosed with atypical scrapie. At present this is a single event, and no conclusion can be drawn about the frequency with which this might happen either experimentally or in the field. With only two animals challenged intracerebrally, and the other recipient remaining negative, it will be impossible to extrapolate whether our finding is truly a stochastic single event, or whether a larger group size would have revealed a trend. The very small group size was an inevitable consequence of the practical limitations of this study given the decision to use only naturally-occurring field case donors, for which material is often sparse and frequently of very poor quality. However, this study provides evidence for the view that conversion/mutation of TSE strains is possible and, irrespective of whether or not such events are stochastic, they can lead to the emergence of potentially zoonotic strains.

There are a number of hypothetical explanations for this unexpected result. The first possibility that had to be excluded was that cross contamination with a CH1641 isolate might have occurred at some point in the experimental procedure. The inoculum was prepared and divided into aliquots on the day of preparation; sheep and mice were challenged using different aliquots of the same homogenate. The only strain identified in mice was atypical scrapie, which was compatible with the diagnosis in the original donor. In addition CH1641 had only been handled by the inoculum preparation team once, two years prior to the inocula for this experiment being prepared, arguing against cross contamination during the preparation of the inoculum. Therefore any possible cross contamination would have had to occur during the inoculation procedure. However, on the inoculation day no CH1641 was handled in the operating theatre—only atypical scrapie, and sterile disposable surgical equipment was used wherever possible and reusable equipment was decontaminated according to strict criteria (one hour immersion in 2M NaOH, followed by autoclaving for 18 mins at 136°C). Additionally, an audit of all prepared CH1641 samples indicated that all the anticipated samples remain in our archive and could not, therefore, have been used in error.

Another possible hypothesis is that the donor sheep might have been co-infected sub-clinically with a CH1641-like classical scrapie strain in addition to atypical scrapie. Such co-infection of different scrapie strains, while observed very rarely, has already been described in field situations [23,37], and in the laboratory context, there is supporting evidence for some heterogeneity of strain populations [38] which may then propagate differently in different hosts. Since the donor animal was an ARR/ARR genotype, this would further reduce the likelihood of a classical scrapie strain co-existing in this sheep, since there are only two confirmed cases of classical scrapie occurring naturally in this genotype [39]. Alternatively, it could be argued that a resistant sheep could potentially be more likely to harbour low levels of a classical scrapie strain which could remain below detection levels of the currently available laboratory tests, given that little is understood about the fundamental basis for genetic resistance. However, surveillance records were reviewed for details relating to the source flock, and there has been no recorded occurrence of classical scrapie in any genotype of animal from this flock, at any time.

The third, and arguably the most plausible, explanation is that the original atypical isolate has ‘converted/mutated’ into CH1641 as a result of the experimental conditions. Again there is some precedent for this occurrence, with previous experimental observations in porcine transgenic mice, where an atypical scrapie isolate acquired a typical 3-band blot pattern with characteristics similar to sub-passaged ovine BSE [40]. This example relates to a cross-species transmission, but evidence also exists of a bovine isolate ‘converting’ or splitting in *PRNP* congenic TgBov mice [26]. If this was to occur in the field it could be speculated that a country such as Portugal, in which, for several years, atypical scrapie was reported in the absence of any classical scrapie, would be a good place to look for evidence of this kind of strain conversion. However, none of the few recently reported classical scrapie cases in Portugal were CH1641-like [41]. Under natural conditions, and assuming that the atypical scrapie agent is shed into the environment at all, animals are most likely exposed to only very small quantities of the agent, over a prolonged period of time, and probably by the oral route. In contrast, in the current study, a significant amount of the agent was inoculated directly to the brain thereby bypassing all potential natural physiological barriers to the disease. This may have put a greater pressure on the agent to propagate and evolve leading to the observed strain conversion. The difference of genotype between donor and recipient sheep may also have contributed to this, although this did not affect the retention of the atypical phenotype in the animal which succumbed to the oral challenge.

It is likely that it will never be possible to determine conclusively what gave rise to this change in phenotype. However, this study adds to the growing body of data which highlights a fundamental problem with prion diseases; in the absence of an agent that is structurally independent form the host genome and which can be isolated and typed, classification of natural disease relies on the phenotypic characteristics of the host. Given that some isolates appear to be unstable under certain circumstances, there is a danger in making assumptions about the disease situation in the field, and the associated public and animal health risks based on observed phenotype alone.

## Supporting Information

S1 File Clinical signs of recipient animal with CH1641 scrapie. A clinical examination carried out at 514 days post inoculation reveals a bilaterally absent menace response: the sheep does not blink in response to movement of a hand/finger towards the eye but blinks when the skin surrounding the eye is touched with artery forceps. Hind limb ataxia is present (excessive swaying of the hind limbs and awkward placement of the limbs when it moves in the corner of the corridor). It bumps its head against the corner of one of the food troughs protruding into the corridor when it turns and also moves once with its head very close to the food trough after it walks from the corner towards the camera, which suggests some visual impairment. It is however able to negotiate obstacles placed on the floor of the corridor without difficulty. Camera surveillance of the pen showed abnormal lying down and rising behaviour of this sheep (identifiable by the orange arrow) compared to its pen mates (identifiable by the white circle).
(MOV)
